# Magnetic Separation Methods for the Detection of* Mycobacterium avium *subsp.* paratuberculosis* in Various Types of Matrices: A Review

**DOI:** 10.1155/2017/5869854

**Published:** 2017-05-31

**Authors:** Marketa Husakova, Radka Dziedzinska, Iva Slana

**Affiliations:** Veterinary Research Institute, Hudcova 70, 621 00 Brno, Czech Republic

## Abstract

The main reasons to improve the detection of* Mycobacterium avium* subsp.* paratuberculosis* (MAP) are animal health and monitoring of MAP entering the food chain via meat, milk, and/or dairy products. Different approaches can be used for the detection of MAP, but the use of magnetic separation especially in conjunction with PCR as an end-point detection method has risen in past years. However, the extraction of DNA which is a crucial step prior to PCR detection can be complicated due to the presence of inhibitory substances. Magnetic separation methods involving either antibodies or peptides represent a powerful tool for selective separation of target bacteria from other nontarget microorganisms and inhibitory sample components. These methods enable the concentration of pathogens present in the initial matrix into smaller volume and facilitate the isolation of sufficient quantities of pure DNA. The purpose of this review was to summarize the methods based on the magnetic separation approach that are currently available for the detection of MAP in a broad range of matrices.

## 1. Introduction

Paratuberculosis (Johne's disease) is a chronic granulomatous enteritis caused by* Mycobacterium avium* subsp.* paratuberculosis *(MAP). The bacterium infects mainly domestic animals such as cattle, sheep, and goats but also wildlife species (e.g., deer) [1]. Animals are usually infected at a young age through contaminated feces, colostrum, and milk or via contaminated feed [2]. The early detection of Johne's disease is difficult because of the long incubation period, which can last 2–10 years before clinical symptoms appear [3]. The progressive stage of infection is characterized by diarrhea and loss of body weight, which can end in death. This results in lower meat and milk yields, and thus paratuberculosis causes significant financial losses to farmers worldwide [1]. MAP is spread mainly through milk and feces [2]. In 2007, 68.1% of U.S. dairy operations were infected with MAP and the National Animal Health Monitoring System released a report highlighting the importance of this disease [4].

Animals with clinical signs represent only a small proportion of the infected animals within a herd. Those with subclinical disease and thus no clinical symptoms can remain undetected for years during which time, as carriers of MAP, they can shed the infectious agent into the external environment [5, 6]. Successful control of paratuberculosis has been difficult because commonly used diagnostic tests are not accurate until the later stage of infection [7, 8].

Different kinds of matrices, such as feces, milk, milk products, colostrum, environmental samples, milk filters, tissue, or blood can be used for the detection of MAP. Feces and milk represent the most commonly tested matrices. However, due to the presence of many inhibitory substances, the usage of extracted DNA from these test matrices can be complicated. In milk and colostrum, fat and high concentrations of milk protein represent the major problem. Redundant substances from feed, phytic acid, and polysaccharides in feces can inhibit DNA amplification [9, 10]. Thus, appropriate sample preparation prior to PCR detection is crucial. For these purposes, new and more sensitive MAP DNA isolation methods based on magnetic separation procedures (immunomagnetic separation and peptide-mediated magnetic separation) have been developed. Commercially available kits with specific reagents remove PCR inhibitors or have greatly improved DNA capture technology by using DNA or cell-binding paramagnetic beads or DNA filters [11].

The aim of this review is to summarize all of the available information regarding MAP cells and DNA isolation methods based on magnetic separation procedures for the detection of MAP from a broad range of matrices.

## 2. Brief Overview of Commonly Used Detection Methods of MAP

There are several methods used for the detection of MAP in infected animals. Cultivation with chemical decontamination was one of the first methods to be developed for MAP detection. Despite the fact that more rapid methods for MAP detection have been reported in recent years,* culture* is still considered to be the “gold standard” even though the method is slow (the incubation of MAP on solid medium lasts at least 3 months), is labour intensive, and has limited sensitivity [12].

The popularity of the* polymerase chain reaction* (PCR) for the detection of MAP has risen in recent years. This method is rapid and sensitive and can be designed very specific for the broad range of different organisms. On the other hand, PCR is very sensitive to the presence of inhibitory substances in samples and when applied directly, sensitivity is very low (especially in milk samples, 23%) [13]. Thus, in any direct PCR the extraction method is a critical step [14]. The majority of PCR protocols for MAP detection target the insertion sequence IS*900*, F*57*,* HspX*, or IS*Mav2* and IS*Map04* [15, 16].

A disadvantage of the PCR methods used for the detection of MAP is that they cannot distinguish between viable and nonviable bacteria in the analyzed samples. For this reason the* phage amplification assay* method was developed. The commercially available FASTPlaque TB™ assay enables rapid detection of viable MAP within 24–48 h based on the count of plaques produced when mycobacteriophage-infected cells burst in a lawn of fast-growing* Mycobacterium smegmatis* [17]. To obtain sufficient specificity, it is necessary to verify the plaques combining the phage amplification assay with another detection method such as conventional PCR [18]. Peptide-mediated magnetic separation (PMS) can be used prior to phage amplification assay to reduce the complexity of the sample and remove the inhibitors which interfere following PCR, necessarily used for confirmation of specificity [19]. PMS-phage assay can be also combined with ELISA consequently called phage-mediated immunoassay [17].

The identification of paratuberculosis can be achieved either through identification of the infectious agent or on the basis of the host's immune response (ELISA) [7]. This antibody detection method enables high-throughput and relatively low-cost analysis. However, the sensitivity of MAP-specific ELISA is generally estimated to be below 50%, depends on the stage of disease, and varies between animal species and the tests that are used [20]. The* gamma-interferon assay* is another immune response detection method used for indirect detection of MAP. This diagnostic test relies on the fact that T-lymphocytes release gamma interferon (INF-*γ*) when exposed to specific antigens, and the test measures the amount of released INF-*γ* [21, 22]. However, as protein purified derivate used for the stimulation of the immune system includes antigens shared with other mycobacteria, false-positive results can occur [23].

## 3. Magnetic Separation Methods

**Figure 1:**
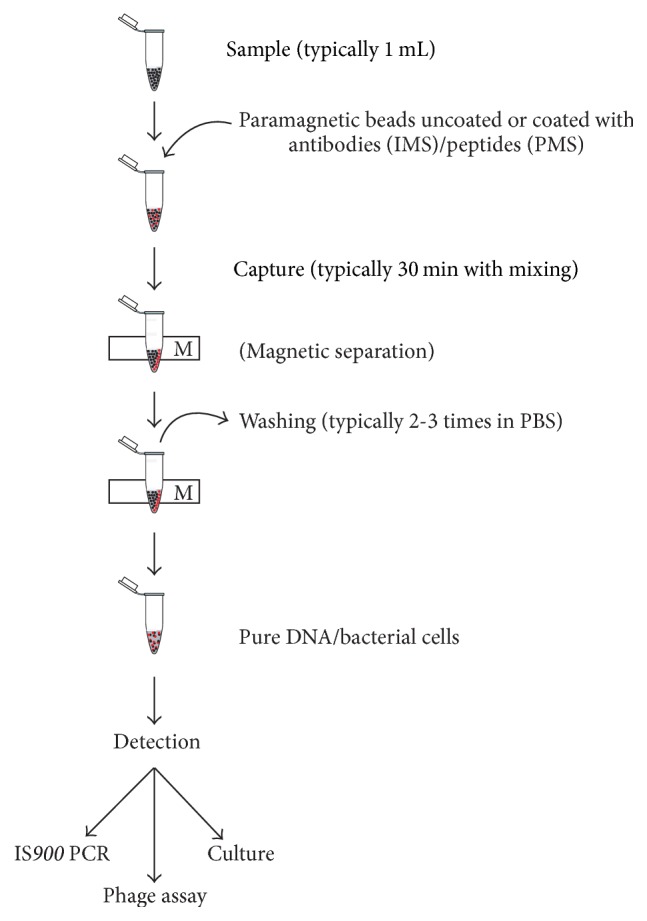
Schematic illustration of the general magnetic separation procedure for the detection of* Mycobacterium avium* subsp.* paratuberculosis*. M, magnetic rack.

Magnetic separation (MS) methods involving either antibodies or peptides (Figure 1) were developed in order to introduce specificity for efficient MAP capture. MS-based methods selectively separate the target bacteria from other, nontarget microorganisms and inhibitory sample components while concentrating the target bacterial cells into a smaller volume. However, Foddai et al. [24] pointed out that not all magnetic separation approaches developed for the selective concentration of MAP perform equally well. The selectivity of capture is assessed by determining the efficiency of capture and depends on the bead characteristics (composition, size, concentration, and surface modification) or the nature of the coating ligand (polyclonal or monoclonal antibody, biotinylated or nonbiotinylated peptide). Capture efficiency, expressed as a percentage, is a measure of the completeness of capture from the original population of target cells present in the sample. Usage of magnetic separation enhances the analytical specificity and sensitivity of the subsequent detection method, which can be culture, PCR, microscopy, an antigen detection immunoassay, or a phage assay [24].

The major weakness of MS-based methods is nonspecific recovery of other* Mycobacterium* spp. Foddai et al. [24] described two types of nonspecific recovery. The first type was observed when uncoated beads were used and the percentage of recovery of nontarget mycobacteria was <10%. This is caused by nonspecific interaction between the surface of paramagnetic beads and bacteria, most likely due to electrostatic bonds or van der Waals forces. The second type of nonspecific recovery, which exceeds 10%, is caused by cross-reaction between the coating ligand (peptide or antibody) and usually closely related nontarget bacteria. Issues with nonspecific recovery could be solved by coating the paramagnetic beads with specific ligands.

Combined MS-based techniques are routinely used for the detection and isolation of pathogenic bacteria such as* Listeria monocytogenes *[25],* Salmonella* spp. [26], and* Escherichia coli* [27] in both food and veterinary clinical samples [28]. In mycobacterial research this approach is still not so commonly used.

MS can be divided, according to the type of capture ligand that is coated onto paramagnetic beads, into immunomagnetic separation and peptide-mediated separation.

The* immunomagnetic separation* (IMS) method relies on the interaction between cell surface antigens and antibodies coated onto paramagnetic beads. The immune part of the beads is represented either by monoclonal or polyclonal antibodies which selectively capture target bacteria. IMS for MAP, first described by Grant et al. [29], employs a rabbit polyclonal anti-MAP antibody, which was used to coat sheep anti-rabbit immunoglobulin G (IgG) type M-280 Dynabeads and facilitates selective isolation of MAP cells from milk. IMS is a powerful method for extracting desired organisms from heterogeneous bacterial suspensions; it shows good detection specificity for MAP and high detection sensitivity, although this is very much dependent on the antibody involved. The speed of MAP detection is enhanced by subsequent IS*900* PCR use. Immunomagnetic PCR (IMS-PCR) can detect as little as 10^3^ CFU/50 mL, which is 1 to 2 log_10_ units lower compared to the number detected by IS*900* PCR applied to milk directly [13]. Use of polyclonal antibody IMS-based methods in conjunction with culture lacks, according to Foddai et al. [24] and sufficient specificity for MAP and nontarget bacteria can overgrow this bacterium in culture. Nevertheless, since no selective medium truly exists, specificity for MAP must be achieved by optimizing the types of beads and capture ligands.

Metzger-Boddien et al. [30] reported a monoclonal antibody IMS-PCR-based method for MAP as a rapid, standardized, and fully automated technique for routine, large scale diagnostic applications. It was shown that this method is very specific and sensitive in detecting minimal amounts of artificially spiked MAP in milk and overall shows superior effectiveness compared to culture.

Most recently, novel monoclonal antibodies for MAP were successfully developed by O'Brien et al. [31]. This new IMS approach uses MyOne Tosylactivated Dynabeads dually coated with monoclonal antibodies 6G11 and 15D10 and promises improvement in sensitivity of MAP capture. However, successful application will be dependent on the end-point detection method and the author suggests to use culture or phage amplification assay.

*Peptide-mediated magnetic separation* (PMS) relies on the interaction between MAP-specific peptides (aMptD, aMp3) coupled with paramagnetic beads and surface-exposed MAP proteins (MptD, Mp3). The 12-mer peptides used were originally identified by phage display biopanning of MAP cells using the Ph.D.-12 phage library and are chemically synthesized to achieve high purity before coating onto paramagnetic beads [32, 33].

PMS can be employed together with other methods such as the* phage amplification assay*. This combination enables rapid enumeration of viable MAP cells within 24 h and is selective for low numbers of MAP. PMS is followed by culture, as a reference method for MAP, but without the need for chemical decontamination [19]. The detection of viable MAP cells and not just MAP DNA is the main advantage of this method over IMS-PCR. Moreover, Foddai et al. [24] reported that the detection sensitivity of the novel automated PMS-phage assay is comparable to or better than those of IMS-PCR methods.

Besides novel monoclonal antibodies, O'Brien et al. [31] generated also new peptide ligands specific for MAP cells and suggested new PMS approach using biotin-EEA402 peptide-coated beads. According to the author, both new IMS and PMS approaches were found to improve the sensitivity of MAP capture compared to the currently used aMp3/aMptD peptide-coated beads. However, these systems are currently under the investigation and need to be applied to matrices to verify their reliable function.

### 3.1. Use of Magnetic Separation Methods for the Detection of MAP Cells

In the following paragraphs, we describe the use of individual types of MS for the detection of MAP. This information is summarized in tables divided according to the type of matrix used. Another partitioning criterion was whether MAP cells or DNA are detected by MS.

### 3.2. Detection of MAP Cells in Milk

The isolation of MAP cells from milk is currently performed using either IMS or PMS methods in conjunction with some end-point detection method, most often IS*900* PCR, phage assay, ELISA, or culture. The most commonly used type of beads for IMS is M-280 sheep anti-rabbit immunomagnetic Dynabeads coated with polyclonal rabbit anti-MAP antibodies (Table 1). Djønne et al. [34] used Dynabeads and IMS-IS*900* PCR for MAP detection in goat milk. The minimal Limit of Detection was determined using unpasteurised cow's milk that was artificially contaminated. Using IMS-IS*900 *PCR, the minimum detection limit for visualization of a product using ethidium bromide was 1 CFU/mL (using dot blot even 0.1 CFU/mL), in comparison with culture where the minimum detection limit was 10 CFU/mL milk. This finding confirms the high sensitivity and great potential of the PCR method for future research. Grant et al. [13] reported 100% sensitivity and 95% specificity of IMS-PCR using Dynabeads coated with polyclonal antibody. In comparison, Metzger-Boddien et al. [30] achieved 96% sensitivity using AnDiaTec ParaTub-S monoclonal antibody-mediated immunomagnetic separation PCR kit in combination with ELISA. This implies that IMS-PCR with Dynabeads is probably the best approach for detection of MAP in milk. Khare et al. [35] used a different type of bead with rabbit polyclonal anti-MAP antibodies, BioMag goat anti-rabbit IgG, with IS*900* qPCR as an end-point detection method. This rapid technique allowed the detection of ten or fewer MAP cells in 2 mL of milk, making this sensitive procedure very useful and cost-effective for the diagnosis of clinical and subclinical paratuberculosis. Recently, Gilardoni et al. [36] combined the use of IMS with monoclonal and polyclonal antibodies and modified IS*900* (IS1 PCR) to detect MAP DNA. The aim of IS1 PCR is to amplify the IS1 fragment in the IS*900* sequence. The IMS-IS1 PCR enabled a Limit of Detection of as low as 10^1^ CFU/mL of milk, when a 50 : 50 mix of monoclonal and polyclonal antibodies was used. This result is better than some of those listed in Table 1, but more investigation of this method is needed.

Dynabeads are the most commonly used beads in PMS studies (Table 1). MyOne Tosylactivated Dynabeads coated with the chemically synthesized MAP-specific biotinylated peptides aMp3 and aMptD were used by Foddai et al. [24]. They achieved 91.5% capture efficiency and minimal (<1%) nonspecific recovery of other* Mycobacterium* spp. In combination with a phage amplification assay. PMS was performed on 1 mL milk samples using 5 *μ*L biotinylated aMp3 peptide-coated beads and 5 *μ*L biotinylated aMptD peptide-coated beads added separately to each reaction. This novel, rapid method for the detection and enumeration of viable MAP cells in milk was proven to be the best available magnetic separation approach, with results obtainable within 48 h.

Subsequent evaluation of the other types of surface-activated paramagnetic beads showed that MyOne Tosylactivated Dynabeads coated with the S624 polyclonal antibody or peptides achieve a much higher capture efficiency for MAP than other surface-activated beads and that, generally, peptide-coated beads consistently achieve higher capture efficiencies than polyclonal-antibody-coated beads. On the other hand, the poorest capture efficiency was recorded for streptavidin-coated beads and amine-coated hollow glass beads, for which mean capture efficiencies were consistently found to be about 10% [24].

The aMptD peptide which binds to the MAP MptD protein was used separately but coupled directly (without a biotin-streptavidin bridge) to paramagnetic beads by carbodiimide in peptide-mediated capture IS*Mav2* PCR [33]. Experiments were based on a competitive capture assay where milk was spiked with MAP; the presence of an excess of other mycobacterial species demonstrated that only MAP had been captured. The results confirmed that the aMptD peptide is highly conserved among different MAP isolates. Independently of the MAP strain, 5 × 10^2^ MAP/mL could be reliably detected. Thus, this demonstrates the high specificity of aMptD and its suitability for use as a ligand in diagnostic tests.

Stratmann et al. [32] reproducibly detected MAP in bulk milk samples using MagneSphere streptavidin paramagnetic beads coated with either phage fMp3 or peptide aMp3 in combination with IS*Mav2* PCR. The Limit of Detection was found to be 10^2^ PFU/mL for fMp3 and 10^1^ PFU/mL for aMp3 peptide. As IS*900* can exhibit nonspecific reactions, IS*Mav2*, present in only three copies in the genome, was used. Foddai et al. [24] could detect as little as 0.3 PFU/mL using Dynabeads, suggesting that it might be interesting to test Dynabeads in conjunction with IS*Mav2* in order to achieve even better results.

### 3.3. Detection of MAP Cells in Feces

The use of magnetic separation for the isolation of MAP from feces is not common. In the literature, only four publications dealing with this technique can be found (Table 2).

Regarding the methods for the isolation of MAP from feces, PMS with Dynabeads is the most commonly used method. However, in one case, IMS using BioMag goat anti-rabbit IgG with rabbit polyclonal anti-MAP antibodies was performed (Table 2).

In all reports bovine feces were used, and, thus, it remains unclear how the method would work for samples other than bovine feces. Frequently, a sample of feces (from 200 mg to 1 g) is diluted and only the suspension is subject to further analysis. Most often, the sample is diluted with water. Only in one case were undiluted feces taken [35, 37].

For the determination of sensitivity and/or LOD (Limit of Detection), artificially contaminated samples were used. Using the IMS method with BioMag goat anti-rabbit immunoglobulin G [IgG] and rabbit polyclonal anti-MAP antibodies, a LOD of 10 or fewer MAP organisms/200 mg of feces was reached, whereas a sensitivity of 100% was achieved [35]. These results show that this procedure is very sensitive and cost-effective for the isolation and detection of MAP cells from feces. Nonetheless, to rigorously verify this method it will be necessary to test naturally contaminated samples, as well as samples of nonbovine origin.

### 3.4. Detection of MAP Cells in Blood

The report of Swift et al. [39] is so far the only available description of the isolation of MAP from clinical blood samples using the PMS-phage method in order to achieve rapid detection of viable MAP (Table 3). To develop the method, 3.5 × 10^1^ PFU/mL of MAP were spiked into commercially available horse or sheep blood. In horse blood, no cells were detectable due to its high viscosity. In initial experiments on sheep blood, only 33% of the cells were recovered. Assuming that the blood was inhibiting the peptide binding or phage assay, samples were subsequently diluted. Samples were diluted 10x in modified 7H9 Media Plus and MyOne Tosylactivated Dynabeads coated with biotinylated peptides (5 *μ*L aMp3, 5 *μ*L aMptD) were used. The PMS-phage method combined with IS*900* PCR was employed resulting in a Limit of Detection of 10 MAP cells/mL of sheep's blood. Moreover, when a 1 in 50 dilution (using modified 7H9 Media Plus) was used for sheep blood, 73% of MAP cells were recovered. However, a 1 in 10 dilution was set as the standard method as it resulted in 92% capture efficiency. These results proved that this assay is rapid and sensitive and can directly detect viable MAP cells present in naturally infected blood, all within 48 h. However, optimization for blood samples from all susceptible animal species is needed.

### 3.5. Detection of MAP Cells in Cheese

It has been found that MAP is a bacterium with extraordinary temperature resistance, and in some cases, it can survive pasteurization of milk and the cheese-manufacturing process. These worrying properties have been verified using several techniques such as conventional cultivation or the plaque assay. However, only one publication reports the use of the MS method in this regard. In 2007, the IMS approach with an automatic Pathatrix magnetic capture system was used to detect MAP in raw bovine milk cheese samples (Table 4). Using several levels of MAP contamination, the LOD was determined to be 10^3^ MAP/25 g. Although no viable MAP cells were detected using conventional cultivation, the use of the F*57*-based real-time PCR system enabled the detection of MAP genetic elements [40].

### 3.6. Use of Magnetic Separation Methods for the Detection of MAP DNA

In case of the MAP DNA detection, paramagnetic beads are not coated with MAP-specific ligands and they do not capture only MAP but also other mycobacterial DNA. Specificity is achieved by end-point PCR method.

### 3.7. Detection of MAP DNA in Milk

As well as the detection of viable MAP cells, direct detection of MAP DNA is also employed. This approach is problematic due to the necessity of obtaining high purity DNA in sufficient amounts for PCR and is complicated by the presence of inhibitors in samples. In order to solve these problems, commercially available kits with DNA capture technology using paramagnetic beads and specific reagents that remove PCR inhibitors have been developed. The study of Donaghy et al. [41] assessed the performance of a commercially available MAP DNA extraction kit for milk, Adiapure, combined with the Adiavet PCR detection kit. Artificially contaminated raw milk with naturally MAP-infected feces (initial number of MAP cells 10^6^–10^7^) was used to assess the sensitivity of MAP detection. The sensitivity of detection was found to be 90%, when 30 copies of IS*900*/mL (equivalent to approximately 2 cells) were detected (Table 5). For 300 copies of MAP, the sensitivity of detection reached 100%. This implies that the commercial Adiapure-Adiavet MAP DNA extraction and PCR detection kit allows consistent and sensitive detection of MAP in milk. In other studies, naturally contaminated milk was used. Herthnek et al. [42] tested the presence of MAP in bulk tank milk using the EZ1 DNA Tissue Kit with paramagnetic beads automatically processed in the BioRobot® EZ1 workstation. The analytical sensitivity was assessed to be 100 MAP/mL for samples of 10 mL (Table 5). Cultured environmental fecal samples (collected from the proximal environment of the cows) were used as a reference and 68% of the herds were positive, while 30% were positive by milk PCR. This result indicates that although MAP may be shed into milk, it will probably be present in low numbers in bulk tank milk due to dilution effects.

### 3.8. Detection of MAP DNA in Feces

Leite et al. [14] compared six commercially available extraction kits for detection of MAP DNA using fecal samples from naturally infected cattle. Using the MagMAX Total Nucleic Acid Isolation Kit containing paramagnetic beads, 76% of the positive samples were identified using IS*900* PCR (Table 6). Using the same extraction kit, 65% of MAP DNA samples were recovered in the study of Okwumabua et al. [43]. According to these results, and in comparison with other nonparamagnetic methods, MagMAX is considered to be a highly efficient method for the extraction of MAP DNA from fecal samples. The BioSprint 96 One-For-All Vet Kit was used by Plain et al. [44] for isolation of MAP from fecal samples from cattle and sheep and from sheep tissue samples. The Limit of Detection was calculated to be 10^2^–10^3^ MAP/mL using artificially contaminated sheep fecal samples with initial concentrations of 10^6^ to 10^1^ MAP/mL.

## 4. General Comments to the Data in Tables

Based on the number of the publications, the most intensively studied matrix using MS is milk. Almost no information is about the use of MS in blood, tissue, and cheese. In case of feces only one type of the origin of matrix was used. Regarding the composition, fecal matrix differs from one animal species to another; thus the access of MS will be also different (depending on the composition of the feed, consistency). There are no publications about the application of MS in infant milk formulas or another animal-derived foodstuff. In the 12th International Colloquium on Paratuberculosis (ICP) in Parma, Italy, there was only one piece of information about the use of MS in infant formulas (page 306; http://www.paratuberculosis.info/images/proc12/12icp.pdf). In the 13th ICP, there was one comparative study on PMS-phage assay with milk suppliers presented (page 48, P-03.10). This presentation highlighted the problem of unsuccessful transfer of the PMS-phage assay method between two different laboratories. Up to date, no publications based on these presentations were published. In addition, there is no investigated environmental matrix (environmental fecal samples from manure, scrapes, etc.). There is a big potential to make the screening of the farm easier with unknown history of paratuberculosis.

In case of all mentioned matrices (except for the tissue) the artificial contamination (AC) with MAP was used. This fact is crucial for the future evaluation. The AC could be used for the exact validation of the method for the selected matrix. Unfortunately, not a lot of publications include important data such as Limit of Detection, capture efficiency, and sensitivity. In general, no sufficient volume of the sample is used. In case of milk, only one milliliter does not have a big informative value and with regard to feces, 200 mg is not sufficient as well.

## 5. Conclusion and Perspectives

Studies published in the last few decades show that the popularity of magnetic separation-based methods has rapidly increased, especially in connection with end-point PCR methods. This trend is due to the fact that MS-based methods are rapid, sensitive, and specific, selectively concentrate target bacteria into smaller volumes, and enable effective removal of PCR-inhibiting compounds. Appropriate sample preparation prior to PCR detection is crucial and for these purposes, new and more sensitive DNA isolation methods have been developed. Obtaining DNA of sufficient concentration and purity for subsequent applications, such as conventional or real-time PCR, represents the main challenges. Therefore, magnetic separation methods remain under development.

The studies discussed in this review document the detection of MAP in milk, feces, cheese, blood, and tissue. Two main approaches are used for the isolation of MAP cells from the above-mentioned matrices, IMS and PMS. For the detection of MAP cells in milk, M-280 sheep anti-rabbit immunomagnetic Dynabeads coated with polyclonal rabbit anti-MAP antibodies are the most commonly used type of beads for IMS. For the detection of MAP cells in feces, the best results are achieved when IMS is used, but PMS with peptide-coated MyOne Tosylactivated Dynabeads is the most commonly employed method at present. PMS in combination with the phage method has been used to determine the viability of MAP cells in blood; other magnetic separation methods have not been used in blood until now. Similarly as in blood, only one study using a magnetic separation method was conducted in cheese until now. Here, the IMS approach with automatic Pathatrix magnetic capture system was applied. For specific capture of MAP DNA, special commercially available kits with paramagnetic beads are used.

An interesting field where the magnetic separation methods were not used until now at all is infant formulas. This could be a field on which the following research should be focused especially when the reports on the presence of MAP in infant formulas appeared recently. In many cases, detection of MAP using magnetic separation methods has been carried out on artificially contaminated samples. For the development of reliable detection methods, more in-depth investigation and the use of naturally contaminated samples, especially of feces, tissues, and blood, is needed. The potential of the magnetic separation methods is broad. However, the main focus of the following research should be to improve the reliability of the assay for the use in many sectors of dairy and food industry.

The novel diagnostic assays for the detection of MAP discussed in this review are giving promising results. Thus far, the assays have not been validated according to OIE recommendations essential for their deployment in the control of animal infectious diseases as well as safeguarding the food chain. Validating the assays according to the OIE rules and testing their robustness in an interlaboratory study would help to harmonize and standardize the variety of assays, increase their acceptance, and facilitate their use in critical studies in animal disease control and food safety.

## Figures and Tables

**Table 1:** Magnetic separation methods used for the detection of *Mycobacterium avium* subsp. *paratuberculosis* cells in milk.

Method	Type of beads	Coating ligand	Type of milk	Artificially contaminated	Preparation of sample	Volume of sample [mL]	Initial number of MAP	Limit of Detection	Capture efficiency (CE) Sensitivity (S)	Reference
IMS	Dynabeads M-280 sheep anti-rabbit IgG^a^	Polyclonal rabbit anti-MAP antibodies	Bovine raw and pasteurized	Yes	Aliquots serially diluted, cultured prior to IMS, or centrifuged and resuspended in PBS before IMS	1	10^6^ CFU/mL	10^4^ CFU/mL	37% (CE)^b^	[29]
			Bovine raw and pasteurized	Yes	No preparation	1, 5, 10, and 50	10^6^ CFU/mL	10^3^ CFU/50 mL	100% (S)	[13]
			Sheep raw	No	No preparation	1	n. a.	n. a.	n. a.	[13]
			Goat raw	No	Milk centrifuged, pellet resuspended in PBS containing 0.05% Tween 20	50	n. a.	n. a.	n. a.	[34]
			Bovine unpasteurised	Yes	MAP suspended in 220 mL milk and diluted 10-fold	50	50 × 10^6^ CFU	1 CFU/mL^c^	n. a.	[34]
								0.1 CFU/mL^d^		
	BioMag goat anti-rabbit IgG^e^	Polyclonal rabbit anti- MAP antibodies	Bovine	Yes	Milk centrifuged, pellet resuspended in PBS	2	10^6^ to 10^0^ organisms/mL	10 or fewer MAP organisms	n. a.	[35]
	ParaTub-S and ParaTub-SL^f^	Monoclonal antibody	Bovine BTM	No^g^	No preparation	1	10^6^–10^0^ organisms/mL	5–10 bacteria/mL^h^ 10–20 cells/mL^i^	96% (S)^j^	[30]
	Goat anti-mouse IgG magnetic beads^k^	Monoclonal anti-MAP antibody 1A6E10 (mAb); polyclonal anti-MAP antiserum (pAb)	Bovine	No^l^	Bacterial clumps broken up by passage through needle, samples heated 15 min at 50°C and centrifuged, pellets resuspended in 1 mL of PBS, and 10 *µ*L of coated beads added	10	10^12^ CFU/mL	10^1^ CFU/mL	n. a.	[36]

PMS	MagneSphere^m^	Biotinylated aMp3 peptide, fMp3 phage	Bovine	Yes	Milk incubated with aMp3 peptide or fMp3 phage coated beads	1	10^0^ to 10^5^ PFU/mL	10^1^ PFU/mL (aMp3);	n. a.	[32]
								10^2^ PFU/mL (fMp3 phage)		
	aMptD peptide paramagnetic beads^o^	aMptD peptide	BTM, pasteurized	Yes	No preparation	1	5 × 10^2^ and 5 × 10^3^ CFU/mL	5 × 10^2^ MAP/mL	5 × 10^2^ CFU/mL (S)	[33]
	MyOne Tosylactivated Dynabeads	Biotinylated peptides (aMp3, aMptD)	UHT	Yes	50 : 50, 5 *µ*L of both types of beads added separately per reaction to 1 mL MB-OADC with MAP	1	10^3^ to 10^5^ CFU	n. a.^p^	91% ± 5% (CE)	[24]
			Bovine BTM	No	Milk centrifuged, pellet resuspended in 1 mL of MB with 10% OADC	50	n. a.	1 to 110 PFU/50 mL	n. a.	[19]
			Bovine UHT	Yes	Serial 10-fold dilutions of MAP in MB with 2 mmol/L CaCl_2_ added (100 *µ*L) to 900 *µ*L aliquots of milk	1	q	n. a.^r^	n. a.	[17]
			Pasteurized	Yes	100 *µ*L of MAP aliquots added to 900 *µ*L of milk, vortexed, and centrifuged, pellets resuspended in 1 mL of 0.05% PBS-Tween20	1	10^4^ CFU/mL	5 × 10^2^ CFU/mL	n. a.	[38]

^a^Dynabeads M-280 sheep anti-rabbit IgG (Dynal, Oslo, Norway); ^b^almost 100% when 10^2^ to 10^4^ of MAP/mL used; ^c^EtBr staining; ^d^Dot Blot; ^e^BioMag goat anti-rabbit IgG with rabbit polyclonal anti-MAP antibodies (Polysciences, Inc., Warrington, Pennsylvania, USA); ^f^the ParaTub-SLand ParaTub-S kits (AnDiaTec GmbH and Co. KG, Kornwestheim, Germany); ^g^artificially contaminated milk was used to set the Limit of Detection; ^h^IMS-real-time PCR; ^i^automated IMS-PCR-ELISA; ^j^ParaTub-S; ^k^goat anti-mouse IgG magnetic beads (New England BioLabs Inc., Ipswich, MA, USA); ^l^artificially contaminated milk was used for standardization of IMS-IS1 PCR; ^m^MagneSphere streptavidin paramagnetic particles (Promega, Madison, Wisconsin, USA); ^o^aMptD peptide coupled directly by carbodiimide method to paramagnetic beads (Chemicell, Berlin, Germany); ^p^the mean LOD_50_ = 14,4 PFU/50 mL equivalent to 0,3 PFU/mL; ^q^the number of PFU mL^−1^ used to spike the samples was determined by subjecting the 10^−3^, 10^−4^, and 10^−5^ spiking dilutions to the optimized phage assay and counting the plaques produced. In the final assay format, a consistent number of D29 seed phages (10^8^ PFU mL^−1^) was added to the samples; ^r^dynamic range of the assay was 3 × 10^2^–6 × 10^8^ phages/mL; BTM = bulk tank milk; CE = capture efficiency; CFU = colony forming units; ELISA = enzyme-linked immunosorbent assay; HEYM = Herrold's egg yolk medium; IMS = immunomagnetic separation; LOD = Limit of Detection; LOD_50_ = 50% Limit of Detection; MAP = *Mycobacterium avium* subsp. paratuberculosis; MB = Middlebrook 7H9 broth; n. a. = not available; OADC = oleic acid-albumin-dextrose-catalase; PBS = phosphate-buffered saline; PFU = plaque-forming units; PMS = peptide-mediated magnetic separation; S = sensitivity; UHT = ultra-heat-treated milk.

**Table 2:** Magnetic separation methods used for the detection of *Mycobacterium avium* subsp. *paratuberculosis* cells in feces.

Method	Type of beads	Coating ligand	Type of matrix	Artificially contaminated	Preparation of sample	Initial volume of sample	Initial number of MAP [MAP/mL]	Limit of Detection	Sensitivity [%]	Reference
IMS	BioMag goat anti-rabbit IgG^a^	Rabbit polyclonal anti-MAP antibodies	Bovine	Yes	MAP aliquots added to 200 mg of feces, resuspended in PBS (2 mL), mixed on a rotating platform, and centrifuged, clear upper phase transferred to a tube with beads	200 mg	10^6^ to 10^0^	10 or fewer MAP/200 mg	100	[35]

PMS	MyOne Tosylactivated Dynabeads	Biotinylated peptides (aMp3, aMptD)	Bovine	No	Sample mixed with 4 mL of sterile water and centrifuged low-speed, 1 mL of supernatant used for PMS	1 g	n. a.	from 6 to 41,1 PFU/g	n. a.	[19]
			Bovine	No	No preparation	2 g	n. a.	n. a.	n. a.	[37]
			Bovine	Yes	Dilution of autoclaved bovine feces in sterile water 1 : 5; serial 10-fold dilutions of MAP prepared in MB containing 2 mmol/L CaCl_2_ and added (100 *µ*L) to 900 *µ*L aliquots of fecal suspension	1 mL	b	from 3 × 10^2^ to 6 × 10^8^ phage mL^−1^	n. a.	[17]

^a^BioMag goat anti-rabbit immunoglobulin G [IgG] with rabbit polyclonal anti-MAP antibodies (Polysciences, Inc., Warrington, Pa.); ^b^the number of PFU mL^−1^ used to spike the samples was determined by subjecting the 10^−3^, 10^−4^, and 10^−5^ spiking dilutions to the optimized phage assay and counting the plaques produced. In the final assay format, a consistent number of D29 seed phages (10^8^ PFU mL^−1^) was added to the samples; IMS = immunomagnetic separation; MAP = *Mycobacterium avium* subsp. *paratuberculosis*; MB = Middlebrook 7H9 broth; n. a. = not available; PBS = phosphate-buffered saline; PFU = plaque-forming units; PMS = peptide-mediated separation.

**Table 3:** Magnetic separation methods used for the detection of *Mycobacterium avium* subsp. *paratuberculosis* cells in blood.

Method	Type of beads	Coating ligand	Type of sample	Artificially contaminated	Preparation of sample	Initial volume of sample [mL]	Limit of Detection [MAP cells/mL]	Initial number of MAP [PFU/mL]	Capture efficiency [%]	Reference
PMS	MyOne Tosylactivated Dynabeads	Biotinylated peptides (aMp3, aMptD)	Sheep	Yes	10-fold dilution of sample in modified 7H9 Media Plus + 5 *µ*L aMp3 and 5 *µ*L aMptD beads	1	10	3.5 × 10^1^	92	[39]
			Horse	Yes		1	n. a.	3.5 × 10^1^	92	
			Bovine^a^	No		1	n. a.	n. a.	92	

^a^Whole blood or isolated buffer coat layer; MAP = *Mycobacterium avium* subsp. *paratuberculosis*. n. a. = information not available; PFU = plaque-forming units; PMS = peptide-mediated magnetic separation.

**Table 4:** Magnetic separation methods used for the detection of *Mycobacterium avium* subsp. *paratuberculosis *cells in cheese.

Method	Type of beads	Coating ligand	Type of matrix	Artificially contaminated	Preparation of sample	Initial mass of sample [g]	Initial number of MAP	Limit of Detection	Reference
IMS	Pathatrix auto system^a^	Anti-MAP antibodies	Bovine raw milk cheese	Yes^b^	Stomacher filter bag, 225 mL of trisodium citrate buffer, 1% casitone, 2% sodium citrate added to the sample, homogenization in a Stomacher 400 lab blender, 50 *µ*L of anti-MAP Pathatrix beads added	25	c	10^3^ MAP/25 g of cheese	[40]

^a^Pathatrix auto system, paramagnetic beads coated with anti-MAP antibody (Matrix MicroScience Ltd., Newmarket, UK); ^b^retail cheese samples included; ^c^different levels of MAP contamination were tested (10^3^  MAP/25 g or 40 MAP/g); auto = automatically processed; IMS = immunomagnetic separation; MAP = *Mycobacterium avium* subsp. *paratuberculosis*.

**Table 5:** Magnetic separation methods used for the detection of *Mycobacterium avium* subsp. *paratuberculosis* DNA in milk.

Type of kit	Type of milk	Artificially contaminated	Preparation of sample	Initial volume of sample [mL]	Initial number of MAP [MAP/mL]	Number of copies detected	Analytical sensitivity [MAP/mL]	Sensitivity of detection [%]	Reference
Adiapure^a^	Bovine raw	Yes^b^	No preparation	10	10^6^–10^7^	30 copies of IS900/mL	n. a.	100, 90, 85, 25^c^	[41]
EZ1 DNA Tissue Kit^d^	Bovine BTM	No^e^	Milk centrifuged, cream retained, pellet incubated (37°C), centrifuged; a mixture of 1000 *µ*L lysis buffer, 2 *µ*L proteinase K used to dissolve the cream fraction, dissolved cream pooled with the pellet, mixture transferred to bead beating tubes (600 *µ*L of beads), tubes incubated (56°C), shaken, centrifuged, 400 *µ*L of the liquid phase processed with kit and BioRobot	10	10^0^–10^4^	n. a.	100	n. a.	[42]

^a^The combined Adiapure-Adiavet MAP DNA extraction and detection kit with magnetic beads; ^b^artificially contaminated with naturally infected feces (10^6^–10^7^ MAP); 10 g thawed feces diluted in 90 mL sterile distilled water; ^c^sensitivity of detection was 100, 90, 85, and 25% for respective MAP concentrations of 300, 30, 3, and 0.3 copies of IS900/mL^−1^; ^d^EZ1 DNA Tissue Kit with magnetic beads (Qiagen, Hilden, Germany) automatically processed in the BioRobot® EZ1 workstation (Qiagen); ^e^for assessment of analytical sensitivity, quantification of MAP and the testing of various parameters during the development of the extraction method artificially contaminated milk was used; auto = automatically processed; BTM = bulk tank milk; MAP = *Mycobacterium avium* subsp. *paratuberculosis*; n. a. = not available.

**Table 6:** Magnetic separation method used for the detection of *Mycobacterium avium* subsp. *paratuberculosis* DNA in feces and tissue.

Type of kit	Type of matrix	Artificially contaminated	Preparation of sample	Initial volume of sample	Initial number of MAP [MAP/mL]	Limit of Detection [MAP/mL]	Sensitivity of detection [%]	Reference
MagMAX^a^	Bovine feces	No	No preparation	500 *µ*L	n. a.	n. a.	65	[43]
		No	300 mg of feces diluted in 1 mL of PBS, vortexed, centrifuged, supernatant added to a tube with zirconia beads and lysis-binding solution, vortexed, centrifuged, added to a plate with isopropanol, magnetic nucleic acid-binding beads and binding lysis enhancer, automated system used (MagMAX Express Magnetic Particle Processor)	300 mg	n. a.	n. a.	76	[14]

BioSprint 96 + MagMAX-96 auto.^b^	Bovine feces	No	Growth in Bactec 12B, 200 *µ*L liquid culture medium added to 200 *µ*L buffer RLT, transferred to a tube with 0.3 g zirconia/silica beads, disrupted in Fast Prep-24 bead beater for 60 s, and centrifuged, 100 *µ*L added to a deep 96-well plate	100 *µ*L	n. a.	n. a.	n. a.	[44]
	Sheep feces	No^c^		100 *µ*L	10^6^ to 10^1^	100–1000	n. a.	
	Sheep tissue	No		100 *µ*L	n. a.	n. a.	n. a.	

^a^MagMAX Total Nucleic Acid Isolation Kit; ^b^BioSprint 96 one-for-all vet kit (Qiagen) + MagMAX-96 automated magnetic processor; ^c^artificially contaminated only to set the Limit of Detection; auto = automatically processed; LOD = Limit of Detection; MAP = *Mycobacterium avium* subsp. *paratuberculosis*; n. a. = not available; PBS = phosphate-buffered saline; RLT = commercial name for the lysis buffer (Qiagen).
